# Understanding the electron pathway fluidity of *Synechocystis* in biophotovoltaics

**DOI:** 10.1111/tpj.17225

**Published:** 2025-01-27

**Authors:** Hans Schneider, Bin Lai, Jens O. Krömer

**Affiliations:** ^1^ Systems Biotechnology Group, Department Microbial Biotechnology Helmholtz Centre for Environmental Research – UFZ Leipzig 04318 Germany; ^2^ BMBF Junior Research Group Biophotovoltaics, Department Microbial Biotechnology Helmholtz Centre for Environmental Research – UFZ Leipzig 04318 Germany

**Keywords:** biophotovoltaics, extracellular electron transfer, pathway fluidity, *Synechocystis*, photosystems, respiration

## Abstract

Biophotovoltaics offers a promising low‐carbon footprint approach to utilize solar energy. It aims to couple natural oxygenic photosynthetic electrons to an external electron sink. This lays the foundation for a potentially high light‐to‐energy efficiency of the Biophotovoltaic process. However, there are still uncertainties around demonstrating the direct coupling of electron fluxes between photosystems and the external electrode. The dynamic cellular electron transfer network linked to physiological and environmental parameters poses a particular challenge here. In this work, the active cellular electron transfer network was modulated by tuning the cultivating conditions of *Synechocystis* and the operating conditions in Biophotovoltaics. The current output during darkness was found to be determined by the intracellular glycogen levels. Minimizing the intracellular glycogen pools also eliminated the dark‐current output. Moreover, our results provide strong evidence that water splitting in photosystem II is the electron source enabling photocurrent, bypassing the microbe's metabolism. Eliminating the storage carbon as possible source of electrons did not reduce the specific photocurrent output, indicating an efficient coupling of photosynthetic electron flux to the anode. Furthermore, inhibiting respiration on the one hand increased the photocurrent and on the other hand showed a negative effect on the dark‐current output. This suggested a switchable role of the respiratory electron transfer chain in the extracellular electron transfer pathway. Overall, we conclude that *Synechocystis* dynamically switches electron sources and utilizes different extracellular transfer pathways for the current output toward the external electron sink, depending on the physiological and environmental conditions.

## Significance Statement

This paper concludes that *Synechocystis* dynamically switches electron sources and utilizes different extracellular transfer pathways for the current output towards the external electron sink, depending on the physiological and environmental conditions. Most importantly, it experimentally demonstrates an efficient coupling of photosynthetic electron flux to the anode, bypassing the microbe's metabolism, as eliminating the storage carbon as possible source of electrons did not reduce the specific photocurrent output.

## INTRODUCTION

The increasing demand for carbon‐free energy production to mitigate climate change and reduce reliance on fossil fuels has sparked the search for innovative and sustainable energy sources. With this perspective, Biophotovoltaics (BPV) has emerged as a promising technology, offering a unique approach to harvesting the power of natural oxygenic photosynthesis for green energy generation (Lawrence et al., 2023; McCormick et al., 2015; Zhu et al., 2023). In BPV photosynthetic microorganisms, such as cyanobacteria or algae, interface with electrodes to generate electrical current or energy carriers (e.g., H_2_). Sunlight is the energy source and water is the electron donor in this process. After billions of years of evolution, photosystems have become highly efficient protein complexes to utilize sunlight for water splitting, with an efficiency of 10–30% (Dau & Zaharieva, 2009), where in contrast the final product of photosynthesis, biomass, typically only conserves about 1% of light energy throughout the entire metabolism necessary for biomass formation. Therefore, compared to other biological or bioelectrochemical systems based on organic carbon derived from biomass, BPVs potentially have a much higher light‐to‐energy efficiency by coupling electron capture directly to photosynthetic electron fluxes originating from light‐driven water splitting. This unique feature positions BPV to be a promising tool for decarbonization of the energy sector, which is the essential measure to realize our climate management goals (Mengis et al., 2022), as well as reducing the land‐use competition of the bio‐energy sector with agriculture.

Over the last two decades, there is an exponentially increasing interest in BPV and other light‐harvesting bioelectrochemical technologies (McCormick et al., 2015). Fundamental research has been conducted in this field, that is, effectively interfacing bacteria with electrodes (Hong et al., 2021), quantitatively analyzing parameters determining power outputs (Bombelli et al., 2011), characterizing extracellular electron transfer (Cereda et al., 2014), or assessing the feasibility of engineering novel extracellular electron transfer pathways (Schuergers et al., 2017). Proof of principle studies have already shown that BPV currents can be used to power small devices like microprocessors continuously for over 6 months (Bombelli et al., 2022) or to facilitate hydrogen evolution at the cathode with a bias of only 0.65 V (Saper et al., 2018). Current production and charge transfer between cells and electrodes, however, are low for autotrophic organisms in BPV compared to the more extensively studied bioelectrochemical systems using heterotrophic organisms (Anam et al., 2021; Nevin et al., 2008; Schneider et al., 2022). Currently, the limited understanding of the underlying electron transfer processes, the so‐called extracellular electron transfer (EET) pathways, is one of the major obstacles for the development and wider application of BPV systems (Bombelli et al., 2011; Lea‐Smith et al., 2016; McCormick et al., 2015).

Cyanobacteria are the preferred photo‐biocatalyst in BPV research due to their simplicity in cellular structure, faster growth, and easier genetic manipulation compared to other photoautotrophs, for example, algae. The cyanobacterium *Synechocystis* sp. PCC 6803 (hereafter *Synechocystis*) is a well‐established model organism for photosynthesis, with a photosynthetic apparatus comparable to that of higher plants (Kaneko & Tabata, 1997; Mills et al., 2020). When illuminated, cells actively concentrate atmospheric CO_2_ as bicarbonate in the carboxysome and convert it to organic carbon with the reducing power supplied by the photosystems. This organic carbon is then used in cellular metabolism to generate biomass and to build up storage compounds, mainly in the form of glycogen (Zilliges, 2014). During darkness, cells are supplied with energy and reducing equivalents through glycolytic breakdown of these intracellular storage pools or organic carbons from the environment and respiratory processes (Cano et al., 2018; Shinde et al., 2020).

**Figure 1:**
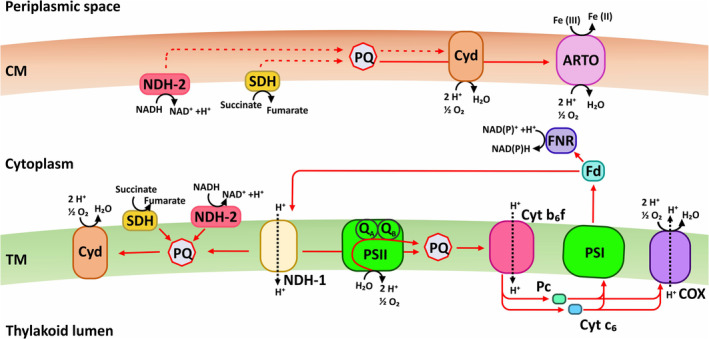
Schematic illustration of cellular electron transport in the membranes of *Synechocystis*. Red arrows indicate direction of the RETC and the PETC, dashed red lines indicate hypothetical electron transport pathways that are not experimentally verified (Lea‐Smith et al., 2016) and black dotted arrows indicate routes of proton transport. ARTO, alternative respiratory terminal oxidase; CM, cytoplasmic membrane; Cox, cytochrome‐c oxidase; Cyd, cytochrome bd quinol oxidase; Cyt b_6_f, cytochrome b_6_f; Cyt c_6_, cytochrome c_6_; Fd, ferredoxin; FNR, ferredoxin NADP+ reductase.; NDH‐1, NAD(P)H dehydrogenase 1; NDH‐2, NAD(P)H dehydrogenase 2; Pc, plastocyanin; PQ, plastoquinone/plastoquinol; PSI, photosystem I; PSII, photosystem II; SDH, succinate dehydrogenase; TM, thylakoid membrane.

*Synechocystis*, like other photoautotrophs, has a complex and dynamic intracellular electron transfer network that is generally categorized into two pathways: the photosynthetic electron transport chain (PETC) and the respiratory electron transfer chain (RETC) (Figure 1). The PETC revolves around two reaction centers: photosystem I and II (PSI and PSII, respectively), localized in the thylakoid membranes (Liberton et al., 2016). A chain of electron carriers consisting of the quinone pool (Q_A_/Q_B_) plastoquinone (PQ), the cytochrome (Cyt) *b*_*6*_ *f* complex, plastocyanin, and ferredoxin connect PSII and PSI (Figure 1) (Lea‐Smith et al., 2016). The RETC in *Synechocystis* encompasses protein complexes located in both thylakoid and cytoplasmic membranes (Peschek et al., 2004). Organic electron carriers such as NAD(P)H or succinate, originating from the breakdown of storage molecules (i.e., glycogen), are oxidized (Cooley & Vermaas, 2001). Electrons are transferred via PQ to Cyt b_6_f, facilitating proton pumping and thus ATP generation. Three terminal oxidases, cytochrome bd terminal quinol oxidase (CYD), cytochrome*‐c* oxidase (COX), and alternative respiratory terminal oxidases (ARTO), transfer electrons to O_2_ as the final acceptor (Howitt & Vermaas, 1998; Lea‐Smith et al., 2013). Shared electron carriers such as PQ, Cyt b_6_f, and plastocyanin indicate a high degree of interconnection between PETC and RETC (Lea‐Smith et al., 2016; Mullineaux, 2014). Along the PETC and RETC pathways, the light energy utilization efficiencies decreased dramatically from over 30% at PSII, to around 11% at PSI and finally to ~1% in the form of biomass (Dau & Zaharieva, 2009; Michel, 2012). Thus, the potential light‐to‐energy efficiency of the BPV process can vary significantly, depending on the molecular mechanism of the EET pathway. It can be either a direct electron transfer of the water‐splitting electrons from the PETC to the anode, or an indirect pathway from PETC to RETC and ultimately to the anode. Both processes could run exclusively or in parallel, upon the physiological and environmental conditions.

To confirm the water‐splitting reaction as the source of photocurrents in BPV, the well‐established PSII inhibitor 3‐(3,4‐dichlorophenyl)‐1,1‐dimethylurea (DCMU) is often used in BPV research (McCormick et al., 2015; Tschortner et al., 2019). DCMU obstructs electron transfer between Q_A_ and Q_B_, thus blocking electron flow from the excited PSII into the PQ pool (Inoué et al., 1990; Trebst, 1987, 2007). By competitively binding to the Q_B_ site in the D1 PSII‐subunit, DCMU blocks the binding site of the native electron acceptor plastoquinone and increases the redox potential of Q_A_ (Broser et al., 2011; Inoué et al., 1990). Multiple studies found a strong reduction of photocurrents produced by *Synechocystis* in BPV systems upon application of DCMU and thus attributed the photocurrent origin to be PSII (Bombelli et al., 2011, 2022; Cereda et al., 2014; Clifford et al., 2021; Hatano et al., 2022; Kusama et al., 2022; Lund et al., 2024; Meng et al., 2021; Zhang et al., 2018). Most studies also showed residual current output after DCMU treatment, which was attributed to the electron flow downstream of PSII that could still contribute toward EET (Bombelli et al., 2011; Cereda et al., 2014; Clifford et al., 2021). Strong inhibition of photocurrents with DCMU was also observed in BPV systems using other cyanobacteria (Gacitua et al., 2020; Pisciotta et al., 2011; Sekar et al., 2014; Torimura et al., 2001), micro‐algae (Beauzamy et al., 2020; Ochiai et al., 1983), macro‐algae (Shlosberg, Krupnik, et al., 2022), intact plant leaves (Shlosberg, Meirovich, et al., 2022), or isolated thylakoids (Calkins et al., 2013; Pinhassi et al., 2016).

However, several studies challenge the general validity of pinpointing BPV photocurrent to PSII after inhibition by DCMU, showing that light‐dependent EET can bypass DCMU inhibition under specific conditions. In BPV systems using the lipid‐soluble redox mediator 2‐hydroxy‐1,4‐naphthoquinone (HNQ), DCMU had only minor influence on the photocurrent. Current outputs of *Anabaena variabilis* in the light are reduced by only 25% in presence of HNQ and O_2_ evolution is reduced by 30–40% (compared to 100% without HNQ) (Tanaka et al., 1988). DCMU reduced HNQ‐mediated photocurrent production from *Synechococcus sp*. by only 20%, suggesting that HNQ accepts electrons mainly at the site before Q_B_ (Yagishita et al., 1993). Specific electrode materials and preparations revealed another mechanism bypassing PSII photocurrent inhibition by DCMU. Thylakoids drop‐casted on RuO_2_‐coated electrodes showed no significant inhibition of photocurrents by DCMU, indicating direct EET (facilitated by physical proximity and independent of a mediator) between thylakoids and the electrode (Hong et al., 2021). Photocurrent production of isolated PSII, adsorbed on the mesoITO, showed a photocurrent and O_2_ evolution to be around 1/3 of the control level. In both cases the authors concluded that electrons are either extracted from the upstream of Q_B_ or directly transferred from Q_A_ to the electrodes *via* direct EET.

Recently published studies also indicate that there are mechanisms for PSII‐independent photocurrent production that are not depending on the application of specific mediators or direct EET. BPV reactors using *Chlorella vulgaris* showed no significant decrease in photocurrent generation in presence of DCMU, compared to controls (Herrero‐Medina et al., 2022). *Synechocystis* cells showed a faster increase and overall higher photocurrents in a mediator‐less BPV system in the presence of DCMU, compared to inhibitor‐free controls (Saper et al., 2018). Furthermore, the authors found that for a PSII‐deficient mutant, DCMU was even essential for photocurrent production (Saper et al., 2018). Another study also confirmed increased photocurrents from *Synechocystis* in the presence of DCMU for a drop‐based BPV system, suggesting production of the photocurrent at secondary sites by either restricting forward electron flow within the respiratory system or by increased release of endogenous electron mediators (Shlosberg et al., 2021).

This paper aims to close this important knowledge gap by providing a deeper understanding of the electron transfer pathways, laying the basis for the optimization of BPV systems. The influence of different electron sources and sinks is investigated in the context of current production in the light and dark by *Synechocystis*. Furthermore, currents are studied both, in the presence and the absence of the commonly used electron mediator ferricyanide. The results reveal that the origin of current production from *Synechocystis* depends on the cellular and experimental conditions, while the addition of the mediator only affects its magnitude. These findings contribute to the understanding of physiological and environmental influences and show that they can drastically change photocurrent production, its origin, and EET pathways.

## RESULTS

### Different light regimes during *Synechocystis* cultivation result in *lush* and *lean* cells

To obtain different physiological states, *Synechocystis* cells with a large glycogen storage pool (*lush* cells) and cells with a depleted glycogen storage pool (*lean* cells) were distinguished. These different cell types were obtained by cultivating *lush* cells under continuous illumination (Figure S1a,c) and *lean* cells under a light–dark cycle (10 h light 14 h dark, Figure S1b,d). The latter condition resulted in slower growth (Figure S1b), smaller cell diameter (Figure S1d) and reduced glycogen accumulation (Figure S2b) compared to *lush* cells (Figure S1a,c; Figure S2a). Cells were harvested after 6–8 days of cultivation resulting in a preculture OD_750_ of ~1 for *lean* cells and ~5 for *lush* cells. At this point, no significant difference in growth rates was observed (Figure S1, 0.17 ± 0.01 day^−1^ for *lush* cells and 0.20 ± 0.01 day^−1^ for *lean* cells, respectively) but a significantly higher glycogen level was present in *lush* cells with 1.7 ± 0.3 pg cell^−1^ compared to *lean* cells with 0.1 ± 0.05 pg cell^−1^ (Figure S2). *Lean* cells showed a continuous decrease of cellular glycogen during dark cultivation with no further degrading after 10–12 h in the dark (Figure S3).

In this study, all BPV experiments were normalized to cell number for better comparison and analysis of current measurements and cellular glycogen content. Normalization to OD_750_ was not considered in this case, as it is influenced by cell diameter, which was significantly different for *lean* and *lush* cells with 2.3 ± 0.12 and 2.9 ± 0.07 μm, respectively (Figure S1c,d). The presentation of current density in regard to (projected) electrode surface was omitted here, since the anode surface chosen ensured that it was not limiting for current transduction.

### Characteristic light current profiles by *Synechocystis* under standard conditions

**Figure 2:**
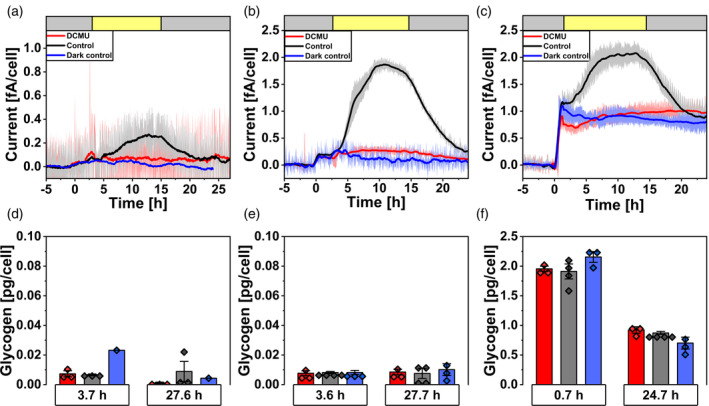
Light current production in the BPV system under standard conditions. (a–c) Chronoamperometry measurements with *lean* (a) *Synechocystis* cells in nBG11without mediator as well as *lean* (b) and *lush* (c) *Synechocystis* cells in nBG11 media with 1 mM ferricyanide as mediator at an applied potential of 0.5 V against the reference electrode. The headspace of the reactors was flushed with air (30 mL min^−1^). Cells were inoculated at 0 h. Red, black, and blue lines represent the smoothed averages of independent measurements (*n* = 3, 3, 1 for experiments without mediator and *n* = 3, 4, 3 for experiments with ferricyanide), with the standard error in the respective color. Yellow and gray bars at the top represent illuminated (50 μmol_photons_ m^−2^ sec^−1^) and dark phases, respectively. Dark controls were not illuminated. (d–f) Glycogen samples for *lean* (d and e) and *lush* (f) cultures were taken at the beginning of the light phase and 24 h later, error bars show the standard deviation between replicates with data points plotted on top of bars.

*Synechocystis* cells showed a characteristic current profile in the BPV system under standard conditions (Figure 2a–c; Figure S4). When cells were inoculated into the BPV reactors containing 1 mM of the redox mediator ferricyanide (hereafter referred to as mediator), dark currents ranging between 0.1 fA cell^−1^ to approximately 1.1 fA cell^−1^ were observed for *lean* cells (Figure 2b) and *lush* cells (Figure 2c), respectively. Upon illumination, the current rapidly increased to around 2 fA cell^−1^ for *lean* and 2.2 fA cell^−1^ for *lush* cells, in the presence of the mediator. During the light–dark cycle in the BPV system, *lean* cells showed no significant changes of the glycogen levels (*P* > 0.05, Figure 2d,e). In contrast, *lush* cells showed a significant decrease of intracellular glycogen from around 2.1 to 0.9 pg cell^−1^ (*P* < 0.001, Figure 2f). The mediator ferricyanide can be considered stable at the applied conditions as observed by photometric measurements with no significant change for over 7 days.

In the absence of the mediator, comparable current profiles at a lower magnitude were observed. Here, initial dark currents increased to 0.06 fA cell^−1^ for *lean* cells (Figure 2a). When illuminated, these reactors showed light currents around 0.3 fA cell^−1^. A similar light current profile was observed for *lush* cells in the absence of the mediator, but with an initial dark current around 0.15 fA cell^−1^ (Figure S4a). No significant glycogen decrease was observed for *lean* cells (*P* > 0.05, Figure 2c), while glycogen levels decreased from 1.6 to 0.5 pg cell^−1^ for *lush* cells (*P* < 0.001, Figure S4b). In all cases illumination had no visible influence on the current profile after the addition of 100 μM DCMU. Currents in the presence of DCMU followed the dark‐current profile or were only slightly higher.

Under standard conditions with ambient levels of dissolved O_2_ and CO_2_, *Synechocystis* cells exhibited a characteristic light‐dependent current profile. In the presence of DCMU, illumination of *Synechocystis* showed no influence on current generation and the current profile followed closely that of unilluminated controls. This current profile is comparable for reactors with and without mediator, while the former showed around 5 times higher peak currents.

### Light currents by *Synechocystis* vary when electron sources and sinks are limited

**Figure 3:**
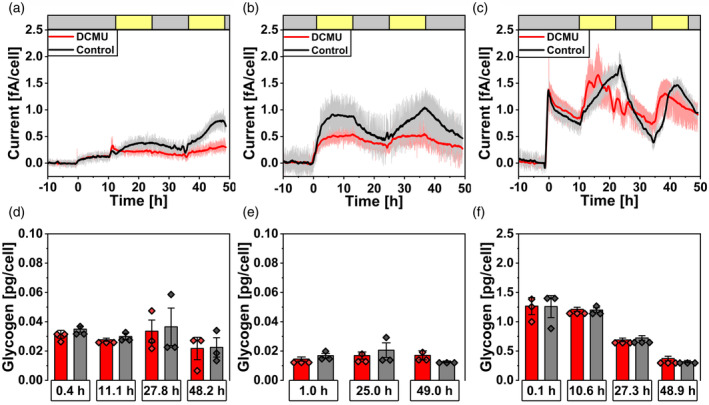
BPV light current production under carbon and O_2_ limited conditions. (a–c) Chronoamperometry measurements with *lean* (a), *intermediate* (b), and *lush* (c) *Synechocystis* cells in carbon‐reduced nBG11 media with 1 mM ferricyanide as mediator at an applied potential of 0.5 V against the reference electrode. The headspace of reactors was flushed with N_2_ (30 mL min^−1^). Cells were inoculated at 0 h. Red and black lines represent the smoothed average of 3 independent measurements each with the standard error in the respective color. Yellow and gray bars at the top represent illuminated (50 μmol_photons_ m^−2^ sec^−1^) and dark phases, respectively. (d–f) Glycogen samples for *lean* (d) *intermediate* (e) and *lush* (f) cultures were taken at the inoculation and at the beginning/end of the first light phase and 24 h later, error bars show the standard deviation between biological replicates (*n* = 3) with data points plotted on top of bars.

Under electron sink‐limited conditions (omitting citrate, carbonate, CO_2_, and O_2_), *Synechocystis* cells exhibited diverse current profiles depending on the level of cellular glycogen and the presence or absence of mediator. When *lean* cells were inoculated into the BPV system (containing mediator) without illumination, an initial dark current close to 0.13 fA cell^−1^ was observed (Figure 3a). When illuminated, a light current around 0.4 and 0.8 fA cell^−1^ was measured for the first or the second light–dark cycle, respectively. Reactors containing 100 μM DCMU indicated only minor influence of illumination on the current with 0.2 and 0.3 fA cell^−1^ for the first and second illumination cycle, respectively.

*Lush* cells exhibited a high dark current which dropped quickly from an initial 1.3 fA cell^−1^ to around 0.8 fA cell^−1^ over 10 h (Figure 3c). When illuminated, the current increased to around 1.9 and 1.7 fA cell^−1^ for reactors without and reactors with 100 μM DCMU, respectively. Under this condition, DCMU‐treated cells showed a distinct, light‐dependent current that increased faster during illumination than the control. This pattern could be reproduced during the second light–dark cycle with currents increasing for controls to 1.5 and 1.35 fA cell^−1^ for reactors with DCMU. Here, *lean* cells exhibited no significant changes of the glycogen levels (*P* > 0.05, Figure 3d) but *lush* cells showed a significant decrease of intracellular glycogen from around 1.4 to 0.4 pg cell^−1^ (*P* < 0.001, Figure 3f).

To validate the results obtained with *lean* and *lush* cells, *Synechocystis* cells were cultivated under continuous illumination but harvested at the early exponential growth phase (OD_750_ ~ 1) resulting in cellular characteristics in between *lean* and *lush* cells without significant change in glycogen (with *P* > 0.05 and values similar to *lean* cells, Figure 3(e), hereafter called *intermediate* cells). When these cells were illuminated immediately after inoculation (Figure 3b), the DCMU‐treated reactors showed a visible light‐dependent current increase to 0.5 fA cell^−1^ (compared to 0.3 fA cell^−1^ for the dark control, Figure S5) compared to 0.9 fA cell^−1^ for illuminated controls. This represents an intermediate state where cells show a light‐dependent current production in the presence of DCMU but at low levels compared to *lush* cells (Figure 3c).

In the absence of the mediator, *Synechocystis* cells showed a similar behavior under carbon and O_2_‐limited conditions (Figure S6a,b). *Lean* cells showed a lower dark current (compared to *lush* cells) and did only exhibit a light‐dependent current increase in absence of DCMU. For *lush* cells, a light‐dependent current for both, controls and DCMU‐treated reactors was observed. Glycogen levels showed no significant change for *lean* cells while *lush* cells exhibited a decrease of intracellular glycogen from 2.1 to 0.8 pg cell^−1^ (*P* > 0.05 and *P* < 0.001 for Figure S6c,d, respectively).

*Synechocystis* cells exhibited diverse current profiles under carbon‐ and O_2_‐limited conditions, depending on intracellular glycogen levels and the presence, or absence, of the mediator. Both *lean* and *lush* cells showed increasing currents during illumination, but *lush* cells demonstrated higher peak currents. In the presence of 100 μM DCMU, *lean* cells exhibited only a minor influence of illumination. In contrast, *lush* cells showed a light‐dependent current increase in the presence of DCMU, both with and without mediator. *Intermediate* cells, which were continuously illuminated in precultures (like *lush* cells) and illuminated upon inoculation into the BPV reactor, showed characteristics between *lean* and *lush* cells regarding current production and DCMU effects.

**Figure 4:**
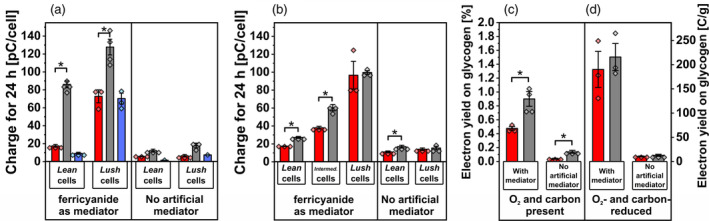
Charge conducted and electron yield in the BPV system with *Synechocystis*. (a–d) Total charge over 24 h for the first light/dark cycle in the BPV system with *Synechocystis* in (a) carbon‐reduced nBG11 flushed with N_2_ or (b) nBG11 media flushed with air. Electron yields (calculated as C g_glycogen_^−1^ and % [C/C] as specified in [Equation 1]) for *lush* cells in (c) reactors with nBG11 media flushed with air and (d) reactors with carbon‐reduced nBG11 flushed with N_2_. Red bars depict DCMU‐treated reactors, and blue bars indicate dark controls. Error bar show the standard deviation between biological replicates (as described in Figures 2, 3, 4; Figures S4 and S6) with data points plotted on top of bars. Asterisks indicate significant differences (*P* < 0.05).

To quantitatively describe this phenomenon, the charge conducted within one 24‐h light–dark cycle was calculated and compared between conditions. In addition, the total charge transferred, and the amount of glycogen consumed were used to estimate an electron yield, describing the share of electrons reaching the electrode that could be explained by glycogen consumption (Figure 4).

All conditions with either *lean* cells (i.e., cells containing low levels of glycogen), or presence of O_2_ and CO_2_ showed significantly decreased charge outputs in the presence of 100 μM DCMU compared to the illuminated controls (Figure 4a). Only for *lush* cells with high glycogen contents, in absence of O_2_ and CO_2_, the addition of DCMU showed no significant difference in charge output (both in presence and absence of mediator, Figure 4b). All experiments with *lush* cells showed a significant decrease of cellular glycogen levels over time, while no significant change for *lean* cells was observed. Thus, the decrease of intracellular glycogen was calculated only for experiments with *lush* cells and compared to the respective charge conducted at the electrode over one light–dark cycle (Equation 1). The electron yield on glycogen varied significantly between experimental conditions (Figure 4c). For experiments with nBG11 and air the electron yield was lower in the presence of 100 μM DCMU with 68 C (Coulomb) g_glycogen_^−1^ compared to 129 C g_glycogen_^−1^ for the controls in the presence of mediator (corresponding to 0.48 and 0.9% electron recovery from glycogen full oxidation, respectively, see Equation 1). In the absence of the mediator, a similar difference in terms of electron yields on glycogen was observed, but values were one order of magnitude lower. In experiments with carbon‐reduced nBG11 and the absence of O_2_ and CO_2_, electron yields reached around 200 ± 13 C g_glycogen_^−1^ (approximately 1.4%) in the presence of the mediator. Here, no significant difference could be observed when 100 μM DCMU was applied (Figure 4d).

### Respiratory dehydrogenase involved in dark‐current output but not photocurrent

**Figure 5:**
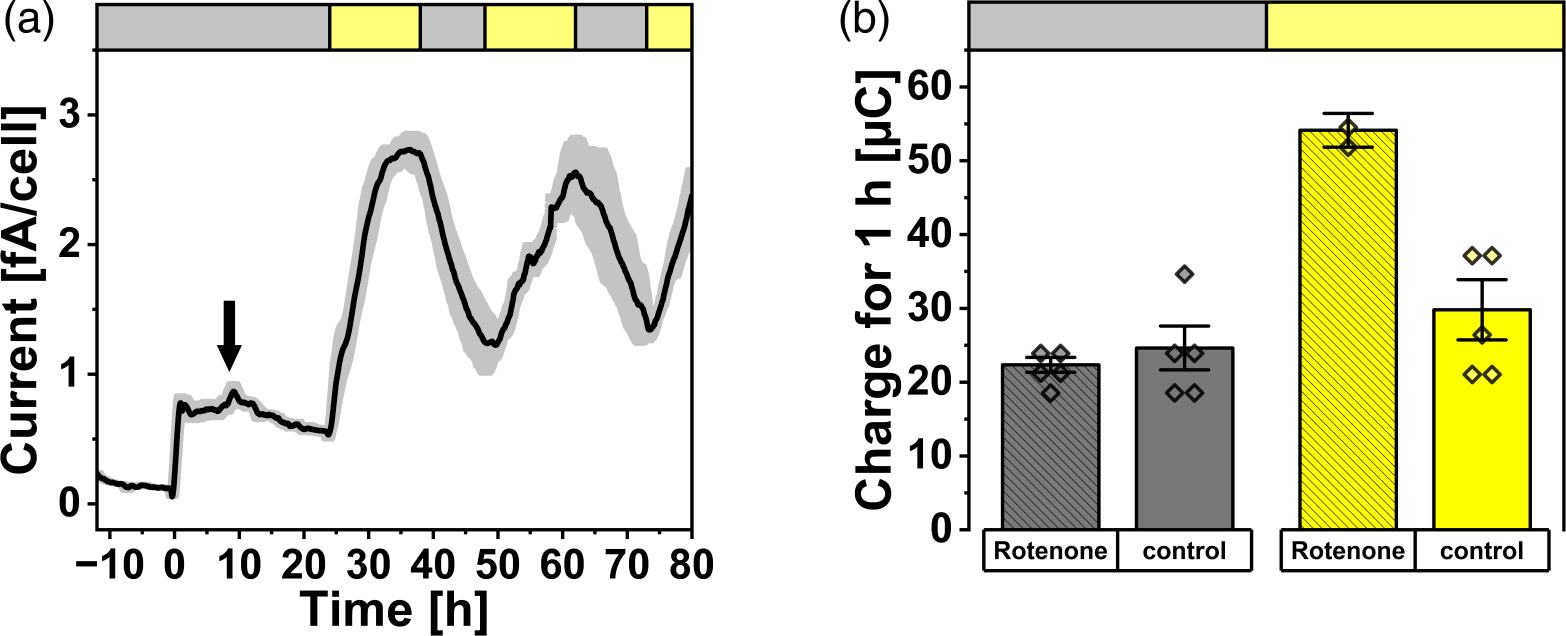
BPV current profile and charge output with the addition of the RETC inhibitor rotenone. (a, b) Chronoamperometry measurements with *lush Synechocystis* cells (a) and comparison of the total charge over 1 h (b) in BPV reactors containing nBG11 media with 1 mM ferricyanide as mediator at an applied potential of 0.5 V against the reference electrode. The headspace of reactors was flushed with air (30 mL min^−1^). Cells were inoculated at 0 h, Rotenone was added 8.5 h later (indicated by black arrow). Black lines represent the smoothed average of 5 independent measurements with the standard error in gray. Yellow and gray bars at the top represent illuminated (50 μmol_photons_ m^−2^ sec^−1^) and dark phases, respectively. The charge for 1 h was calculated from absolute current measurements and not normalized to cell number with the light conditions based on the table 2 from Schneider et al. (2023), with data points plotted on top of bars.

To further investigate the pathway of respiratory electrons to EET the RETC inhibitor rotenone was added to BPV experiments with *lush* cells during the initial dark phase (Figure 5). After establishment of a current around 0.73 fA cell^−1^ in the absence of illumination, the addition of rotenone resulted in a small current increase to around 0.87 fA cell^−1^ followed by a decrease of the dark‐current progression to 0.53 fA (Figure 5a; Figure S7). Subsequent illumination of the system showed a typical light‐induced current profile but with higher peak currents (2.7 fA cell^−1^) compared to experiments without rotenone (Figure 5a). The average charge output of the BPV system (for 1 h) in the dark was 24.6 μC compared to 22.3 μC after the addition of rotenone (Figure 5b). Previous investigations showed a significantly higher charge output of *Synechocystis* by the addition of rotenone under illumination with 49.2 μC compared to 31.2 μC without the addition of rotenone [calculated for 1 h, see Schneider et al. (2023, table 2)].

### PSI activity is influenced by cellular and experimental condition

**Figure 6:**
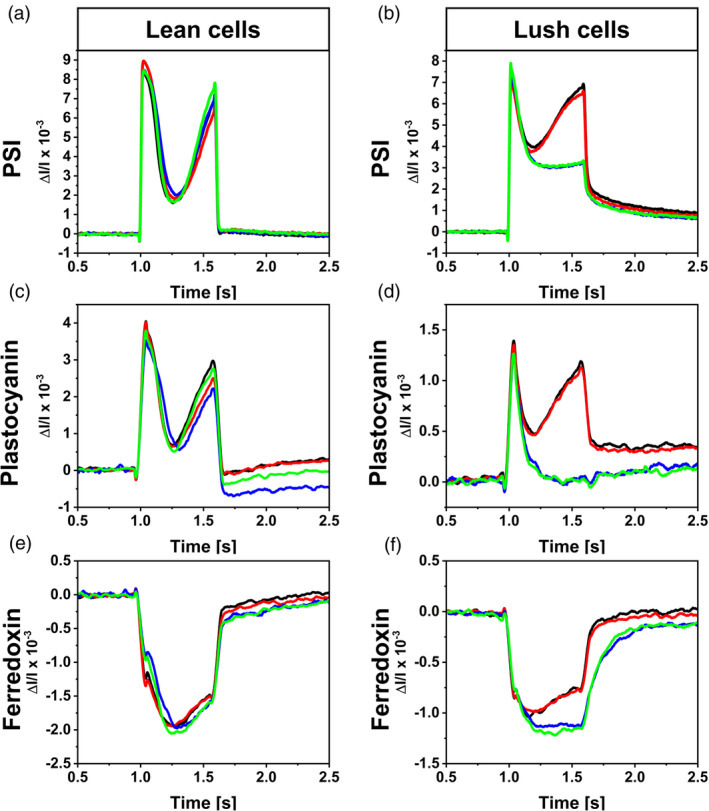
Model spectra of *Synechocystis*. (a–f) Redox changes of PSI, plastocyanin, and ferredoxin for *Synechocystis lean* cells (a, c, e) or *lush* cells (b, d, f). Cells were resuspended in nBG11 media (black), nBG11 with 1 mM of the mediator ferricyanide (red), carbon‐reduced nBG11 media (blue), or carbon‐reduced nBG11 with 1 mM of the mediator ferricyanide (green). Samples with carbon‐reduced nBG11 media were bubbled with N_2_ before each measurement. At 1 sec, an actinic light pulse of 1350 μmol_photons_ m^−2^ sec^−1^ was applied for 600 ms. The traces show the smoothed average of 24 measurements (*n* = 3 biological replicates * 8 technical repeats) with 30 sec dark intervals in‐between.

To test if the availability or limitation of electron donors (i.e., glycogen, citrate, PSII) or electron acceptors (i.e., O_2_, mediator, CO_2_) would lead to differences in electron flow through PSI, *Synechocystis* absorption changes were measured *via* a DUAL‐KLAS‐NIR fluorometer (Figure 6). The DUAL‐KLAS‐NIR monitors changes in near‐infrared absorption by measuring four separate wavelength pairs. This enables the extraction of contributions from PSI, plastocyanin, and ferredoxin from the observed signals (Klughammer & Schreiber, 2016). In these measurements positive values indicate oxidation and negative values indicate the reduction of the respective pool.

The DUAL‐KLAS‐NIR measurements indicated a comparable PETC electron flow for all conditions with *lean* cells. No significant influence of the media type or O_2_ and CO_2_ availability was observed (Figure 6). For *lush* cells in carbon‐reduced nBG11 media saturated with N_2_, no re‐oxidation of PSI and plastocyanin was observed after the initial oxidation with the light pulse. The same cells showed a similar re‐oxidation pattern as *lean* cells in presence of O_2_ and CO_2_. Only minor deviations were observed between presence and absence of the mediator.

## DISCUSSION

The intracellular electron transfer network of *Synechocystis* (Figure 1) allows the cells to reroute electrons dynamically in response to changing environmental parameters, such as varying insolation, or their physiological state. While all electrons ultimately originate from H_2_O splitting in a photoautotroph, the point of electron extraction into the EET is a critical parameter for energy production in BPV, as it defines the overall light‐to‐energy efficiency of the process. Our previous work found many of the commonly used photosynthetic and respiratory electron transfer inhibitors show strong interference with the electrochemical measurement (Schneider et al., 2023), and thus it is difficult to apply such an approach to systematically investigate the relevance of each PETC and RETC component to the EET pathway. Instead, by varying the duration and illumination of the preculture in this work, *Synechocystis* cells with significant differences in their storage glycogen content (Figures S1 and S2) were obtained. This allowed investigations of EET fluxes from water splitting directly versus electron fluxes rerouted from carbon metabolism.

The prevailing hypothesis in BPV research states that light currents by *Synechocystis* originate mainly from PSII and dark currents depend on respiratory electrons (Bombelli et al., 2011, 2022; Cereda et al., 2014; Clifford et al., 2021; Hatano et al., 2022; Kusama et al., 2022; Meng et al., 2021; Tanaka et al., 2021; Zhang et al., 2018). This is true under “standard conditions” (nBG11 media with O_2_ and CO_2_ present), here dark currents are determined by cellular energy storage levels, with glycogen serving as an indicator (Figure 2). This supports previous reports, that is, by Tanaka et al. demonstrating that EET current in *Synechocystis* can be also generated through dissimilation of glucose (Tanaka et al., 2021). However, our results further demonstrate that light current amplitudes remain consistent regardless of glycogen levels, showing similar values for both *lush* and *lean* cells. Therefore, peak currents during illumination thus reflect the maximum EET rate for the respective condition, which is independent of the cellular glycogen level. The presence or absence of mediator results in a comparable current pattern, though with different magnitudes. The EET rate is approximately five times higher when the mediator is present. The photocurrent output pattern further indicates that electrons should directly come from the photosynthetic water splitting, while the storage carbon is not an essential contributor. In fact, this study (Figure 5), as well as previous work (Schneider et al., 2023) found that photocurrent outputs even increased when respiration is inhibited by the addition of NDH‐dehydrogenase inhibitor rotenone. Overall, it is demonstrated that the photosynthetic electron flux can directly and efficiently flow toward the mediator, without going *via* carbon metabolism and respiration.

The overall higher charge output from *lush* cells indicates that ATP and reducing equivalents from respiration are adding to photosynthetically produced electrons in the conducted current. Previous reports have indicated that ATP, provided from endogenous glycogen, is required to drive photosynthetic electron transport to produce current (Yagishita et al., 1993). When cells are treated with DCMU under standard conditions, the current progression and transferred charge resemble dark controls, highlighting that under these conditions PSII is primarily responsible for the light current. This also indicates no significant difference in cyclic electron transport or terminal oxidase activity between *lean* and *lush* cells. Reports showed that wild‐type *Synechocystis* cells display a high capacity of cyclic electron transport around PSI via the NDH which ensures the reduction of the ETCs even when electron donation by PSII is prevented by DCMU (Mi, Endo, Schreiber, & Asada, 1992; Mi, Endo, Schreiber, Ogawa, & Asada, 1992; Theune et al., 2021). In the presence of DCMU, the exhaustion of NADPH is observed, which is consumed by the Calvin cycle and thus withdrawn from cyclic electron transport (Mi et al., 2000).

The assumption that the origin of current is dynamically switched and alternative routes can fuel EET (Saper et al., 2018; Shlosberg et al., 2021) is shown by the distinct effect of rotenone on photocurrent and dark‐current outputs. The increased photocurrent output indicated a competition of respiration with the EET pathway (Schneider et al., 2023), while the effect of rotenone on dark current suggested that the NDH‐dehydrogenase would be very likely involved in the EET pathway in the dark phase (Figure 5). Such a conclusion could be further supported by the cases where electron acceptors in the BPV system are limiting. Under these conditions, when carbon‐reduced nBG11 was used without O_2_ or CO_2_ present, light and dark currents varied based on cellular glycogen storage levels, showcasing lower photocurrents for low glycogen and vice versa. This is observed in both the presence and absence of the mediator, with lower magnitudes for the latter. Under these conditions, *lush* cells show a light‐dependent current response indicating an interaction between photo‐induced currents and respiratory electron availability. The limitation of O_2_ and carbon is deemed relevant for facilitating a PSII‐independent photocurrent. The DCMU‐treated *lush* cells presented a light‐dependent current output, at a level that was similar to the controls (Figures 3 and 4). Similar behavior was observed for a PSII‐deficient mutant of *Synechocystis*, which has shown photocurrent production matching that of the wild type, indicating that PSII is not essential for photocurrent production under the applied conditions (Saper et al., 2018). The DUAL‐KLAS‐NIR results indicated a decreased electron flow downstream of PSI (Figure 6). This suggested PSI acceptor side limitation can be explained by a reduced Mehler‐like reaction or terminal oxidase activity for *lush* cells when O_2_ is limiting. Simultaneously, the PQ pool reduction by glycogen in combination with a low NADP+/NADPH ratio could further limit the plastocyanin, PSI, and ferredoxin oxidation. These results suggested that a PSI‐centric EET mechanism was present for the *Synechocystis* cells with the absence of metabolic electron sinks in BPV, that is, the respiratory electrons from degradation of carbon storage compounds reduced the PETC/RETC shared PQ pool and then transferred to PSI before reaching the EET pathway.

This report shows that photocurrents can originate from PSII as well as PSI, depending on the cellular and environmental conditions. These results join both: the prevailing findings that *Synechocystis* photocurrents originate mainly from water splitting at PSII (Bombelli et al., 2011, 2022; Cereda et al., 2014; Clifford et al., 2021; Hatano et al., 2022; Kusama et al., 2022; Meng et al., 2021; Zhang et al., 2018) as well as more recent results by Saper et al. suggesting light currents can be facilitated by a different electron source, involving the PQ pool, with PSI as the site of photoactivity (Saper et al., 2018).

Dark‐current outputs reveal the availability of respirational electrons is closely linked to the level of intracellular glycogen storage. While *lush* cells have a high capacity of electrons available in the dark, showing high EET rates, *lean* cells can only produce minor dark currents. The same current profiles are observed for these cells under standard conditions when DCMU is added, confirming glycogen as an electron source for the current. The same photocurrent rate is observed for these conditions showing that photocurrents are not just an addition of a fixed rate onto the dark‐current production, but a complex interaction of different electron sources and sinks.

**Figure 7:**
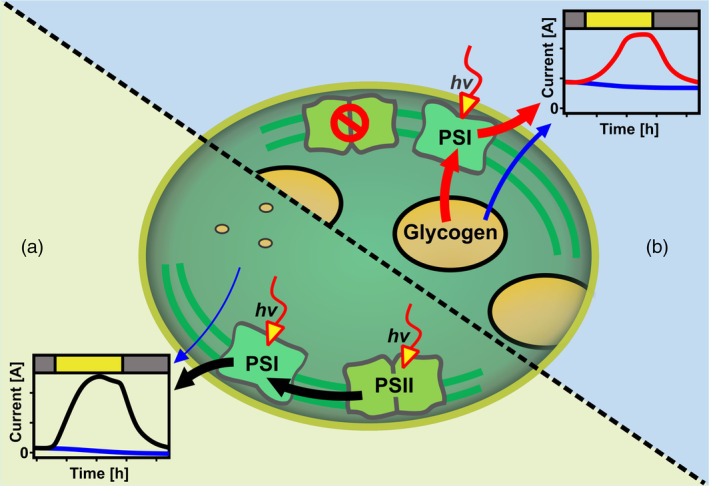
Dynamic model for EET by *Synechocystis* in a BPV reactor. (a, b) Schematic depiction of *Synechocystis* EET pathways and respective electron sources for (a) *lean* cells under standard conditions and (b) *lush* cells, when PSII is not contributing to EET and electron acceptors are limiting.

We suggest a highly dynamic model for electron sources of photocurrents that can switch depending on the availability of extra‐ and intracellular electron sources and sinks (Figure 7). Here, cells with low respiratory electron storage can only achieve high EET rates when PSII water splitting is active under standard conditions (Figure 7a). In the absence of O_2_, CO_2_, and organic carbon cells show lower photocurrents, which could be explained by impairment of other cellular processes like biomass formation or the repair of cellular components. When respiratory electrons are not available (i.e., minimal glycogen storage) cells are unable to produce a photocurrent under these conditions if PSII is inhibited. On the other hand, when respiratory electrons are highly abundant for the cells, *Synechocystis* can produce photocurrents with high EET rates even if PSII is inhibited and the environment is absent of O_2_, CO_2_, and organic carbon (Figure 7b). Here a lower EET rate is only observed for the case when PSII is inhibited and terminal oxidase rates are not restricted by O_2_ availability.

With this work, we connect and clarify existing theories about the origin of currents in BPV and show that EET pathways can dynamically switch depending on the experimental conditions in the context of cellular physiology. The coupling of the photosystem to the EET without the involvement of carbon turnover and respiration was solidly confirmed in this paper, which demonstrated the promising high light‐to‐energy potential of the BPV technology. This research closes a significant knowledge gap and is a major step toward understanding the basis of EET, which is essential for the design and development of BPV as a renewable energy source in relevant scales for humanity.

## MATERIALS AND METHODS

### Strain, medium, and growth conditions

*Synechocystis* sp. PCC6803 wild‐type (Pasteur Culture Collection, Paris, France) cultures were inoculated on BG11 medium (Table S1, supplemented with 0.75% (w/v) agar and 0.03% (w/v) sodium thiosulfate) from cryo‐conserved stocks. Liquid cultures were inoculated into nBG11 media (Table S1, 50 mL in 250 mL baffled Erlenmeyer flasks with cotton plugs) and cultivated in a photo‐incubator (Multitron Pro, INFORS HT, Bottmingen, Switzerland) at 150 rpm (25 mm orbit), 30°C, 75% relative humidity, 50 μmol_photons_ m^−2^ sec^−1^ (cool white LEDs) and ambient CO_2_. Cell concentrations were measured with a spectrophotometer (Libra S11, Biochrom, Cambridge, United Kingdom) *via* optical density at 750 nm (OD_750_) with water as blank (no significant difference compared to media). Cell number and size were measured with a Coulter counter (Multisizer, Beckmann Coulter, Brea, USA).

To obtain *Synechocystis* cultures with low levels of intracellular glycogen (*lean* cells), cells were cultivated under a 10 h light–14 h dark cycle for approximately 7 days and harvested at the end of the dark cycle when they reached the linear growth phase (OD_750_ ~ 1). For high glycogen‐containing cells (*lush* cells), cultures were incubated for around 7 days under continuous illumination until the late linear growth phase (OD_750_ ~ 5). Cells were harvested by centrifugation (5000×*g*, 25°C for 5 min), resuspended in fresh medium, and injected into the reactor using a sterile syringe.

### BPV setup and experiment parameters

All electrochemical measurements were conducted in a lab‐scale BPV system with a three‐electrode setup as described by Lai et al. (2021). In brief, the reactor has a working volume of 220 mL in the anode chamber with carbon cloth, pretreated with 2 mM CTAB (Lai et al., 2016), as working electrode. nBG11 medium or a carbon‐reduced variant thereof was used as electrolyte (Table S1). The headspace of the reactors was flushed either with 30 mL min^−1^ sterile air for nBG11 or N_2_ for experiments with carbon‐reduced nBG11. Chronoamperometry measurements were conducted at a bias potential of 0.697 V against the SHE (reference electrode: Ag/AgCl/KCl_sat_ [Cat. 013457, RC‐1CP, Als, Tokyo, Japan], +197 mV vs SHE) using a potentiostat (VSP, BioLogic, Seyssinet‐Pariset, France). 1 mM Potassium hexacyanoferrate (III) (13746–66‐2, ≥99% purity, Sigma/Merck, Darmstadt, Germany) was used as an artificial electron mediator (hereafter called “mediator”), as specified. Stainless steel (3 × 7 cm mesh, Advent Research Material, Oxford, England) connected to a titanium wire served as counter electrode, separated from the anode chamber via a proton exchange membrane (PEM, Membranes international, Ringwood, USA).

Reactors were inoculated with cells to a final OD_750_ of ~0.8, corresponding to approximately 3 × 10^7^ cells mL^−1^. The PSII inhibitor DCMU (330‐54‐1, ≥98% purity, Sigma/Merck, Darmstadt, Germany) was dissolved in 200 μL ethanol and added to the reactors via a sterile syringe to a final concentration of 100 μM (controls were injected with the same volume of pure ethanol). The RETC inhibitor rotenone was added likewise, but with 300 μL ethanol to a final concentration of 250 μM.

### Calculation of current and charge

Current values from BPV chronoamperometry measurements were normalized to cell number and graphed as smoothed average of independent replicates. All graphs were prepared with OriginPro2023 and statistics were obtained using the integrated One‐Way‐ANOVA test with a significance level of *P* < 0.05. To calculate the charge output, normalized currents were summed up over the first light–dark cycle (24 h). Electron yields from glycogen degradation were calculated with the following equation:
(1)

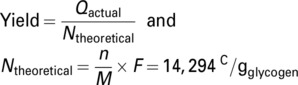




With *n* = 24 mol (number of electrons release from the oxidation of 1 mol glucose to CO_2_), *M* = 162 g mol^−1^ (molar mass of glucose moiety in glycogen), and *F* = 96 485 C mol^−1^ (Faraday constant).

### Cellular pigment determination

For cellular chlorophyll a (Chl_A_) and carotenoids measurements, the protocol developed by Zavřel et al. (2015) was applied. In brief, 1 mL of cell suspension was centrifuged (17 000*g*, 4°C, 10 min) and the supernatant was removed thoroughly. The pellet was resuspended in 1 mL cold (4°C) methanol and incubated at 4°C for 20 min. The absorbance of the supernatant was measured at 470, 665, and 720 nm. The concentrations of Chl_A_ and carotenoids were calculated with the following equations (Ritchie, 2006; Wellburn, 1994):

(2) $${\mathrm{Chl}}_{A}\;\left[\mathrm{\mu g}*{\mathrm{mL}}^{-1}\;\right]=12.9447\;\left(\lambda_{665}-\lambda_{720}\right)$$

(3) $$\;\text{Carotenoids}\left[\mathrm{\mu g}*{\mathrm{mL}}^{-1}\right]=\frac{\left[1000\left(\lambda_{470}-\lambda_{720}\right)-2.86\left({\mathrm{Chl}}_{A}\left[\mathrm{\mu g}*{\mathrm{mL}}^{-1}\right]\right)\right]}{221}$$

### Intracellular glycogen measurement

Samples were collected from *Synechocystis* liquid cultures or BPV reactors, centrifuged (17 000*g*, 10 min, 4°C), and stored at −20°C. Cells were lysed with 30% (w/v) KOH and glycogen was precipitated and washed with ethanol (purity ≥99%). Measurement was conducted *via* Glycogen Assay Kit (Sigma Aldrich, Darmstadt, Germany) according to the manufacturer's instructions with fluorometric readout using a M200 Pro (Tecan Group, Männedorf, Switzerland) plate reader. Total glycogen contents of BPV reactors were then normalized to reactor volume and cell concentration to obtain the glycogen content per cell.

### DUAL‐KLAS‐NIR measurement

The DUAL‐KLAS‐NIR (Heinz Walz GmbH, Effeltrich, Germany) is a compact measuring system facilitating the *in vivo* assessment of transmittance changes in the near‐infrared that can be deconvoluted to redox changes of P700 (PSI), plastocyanin, and ferredoxin (Klughammer & Schreiber, 2016). For all measurements liquid cultures were concentrated by centrifugation (3000*g*, 25°C, 5 min), resuspended in fresh nBG11 or carbon‐reduced nBG11 (saturated with N_2_) media. Measurements were conducted in the DUAL‐KLAS‐NIR with a quartz cuvette containing 1.5 mL cell suspension (correlating to a Chl_a_ concentration of 20 μg mL^−1^). Model spectra of *Synechocystis* were obtained with an actinic light pulse of 1350 μmol_photons_ m^−2^ sec^−1^ applied for 600 ms (Theune et al., 2021). Measurements were repeated 8 times, with 30 sec dark intervals in‐between, for 3 replicates each. Results show the smoothed average of 24 measurements (*n* = 3) for each condition.

## AUTHOR CONTRIBUTIONS

Conceptualization: HS, BL, JOK. Methodology: HS. Investigation: HS. Visualization: HS. Supervision: BL, JOK. Writing—original draft: HS. Writing—review & editing: HS, BL, JOK.

## CONFLICT OF INTEREST STATEMENT

The authors declare that they have no competing interests.

## Supporting information


**Figure S1.** Growth and cell size of *Synechocystis* cultivated under different illumination cycles.
**Figure S2.** Chlorophyll, carotenoid and glycogen content of *Synechocystis* liquid cultures.
**Figure S3.** Growth, Chl_A_ and glycogen content of *Synechocystis lean* cells during dark phase.
**Figure S4.** BPV light current production and in the BPV system without mediator under standard conditions.
**Figure S5.** BPV dark current production under carbon and O_2_ limited conditions.
**Figure S6.** BPV light current production under carbon and O_2_ limited conditions.
**Figure S7.** Chronoamperometry measurements with *lush Synechocystis* cells with the addition of the RETC inhibitor rotenone.
**Table S1.** Constituents and concentrations in BG11, nBG11, and carbon‐reduced nBG11 media.

## Data Availability

All data needed to evaluate the conclusions in the paper are present in the paper and/or the Supplementary Materials. Raw data will be available on request.
