# Supplementary material for: Understanding the electron pathway fluidity of Synechocystis in biophotovoltaics
Source: Plant J. 2025 Jan 27;121(2):e17225. doi: 10.1111/tpj.17225 (PMC11771661; doi:10.1111/tpj.17225)
Supplement: Supplementary file 1 — Figure S1. Growth and cell size of Synechocystis cultivated under different illumination cycles. Figure S2. Chlorophyll, carotenoid and glycogen content of Synechocystis liquid cultures. Figure S3. Growth, ChlA and glycogen content of Synechocystis lean cells during dark phase. Figure S4. BPV light current production and in the BPV system without mediator under standard conditions. Figure S5. BPV dark current production under carbon and O2 limited conditions. Figure S6. BPV light current production under carbon and O2 limited conditions. Figure S7. Chronoamperometry measurements with lush Synechocystis cells with the addition of the RETC inhibitor rotenone. Table S1. Constituents and concentrations in BG11, nBG11, and carbon‐reduced nBG11 media. [file TPJ-121-0-s001.docx]

Supplementary Materials for

**Understanding the electron pathway fluidity of Synechocystis in Biophotovoltaics**

Hans Schneider, Bin Lai,^*^ Jens O. Krömer

*Corresponding author. Email: [bin.lai@ufz.de](mailto:bin.lai@ufz.de)

**This PDF file includes:**

| Item | Description |
| --- | --- |
| Figure S1 | Growth and cell size of *Synechocystis* cultivated under different illumination cycles. |
| Figure S2 | Chlorophyll, carotenoid and glycogen content of *Synechocystis* liquid cultures. |
| Figure S3 | Growth, Chl_A_ and glycogen content of *Synechocystis* *lean* cells during dark phase |
| Figure S4 | BPV light current production and in the BPV system without mediator under standard conditions. |
| Figure S5 | BPV dark current production under carbon and O_2_ limited conditions |
| Figure S6 | BPV light current production under carbon and O_2_ limited conditions. |
| Figure S7 | Chronoamperometry measurements with *lush* *Synechocystis* cells with the addition of the RETC inhibitor rotenone. |
| Table S1 | Constituents and concentrations in BG11, nBG11, and carbon-reduced nBG11 media. |


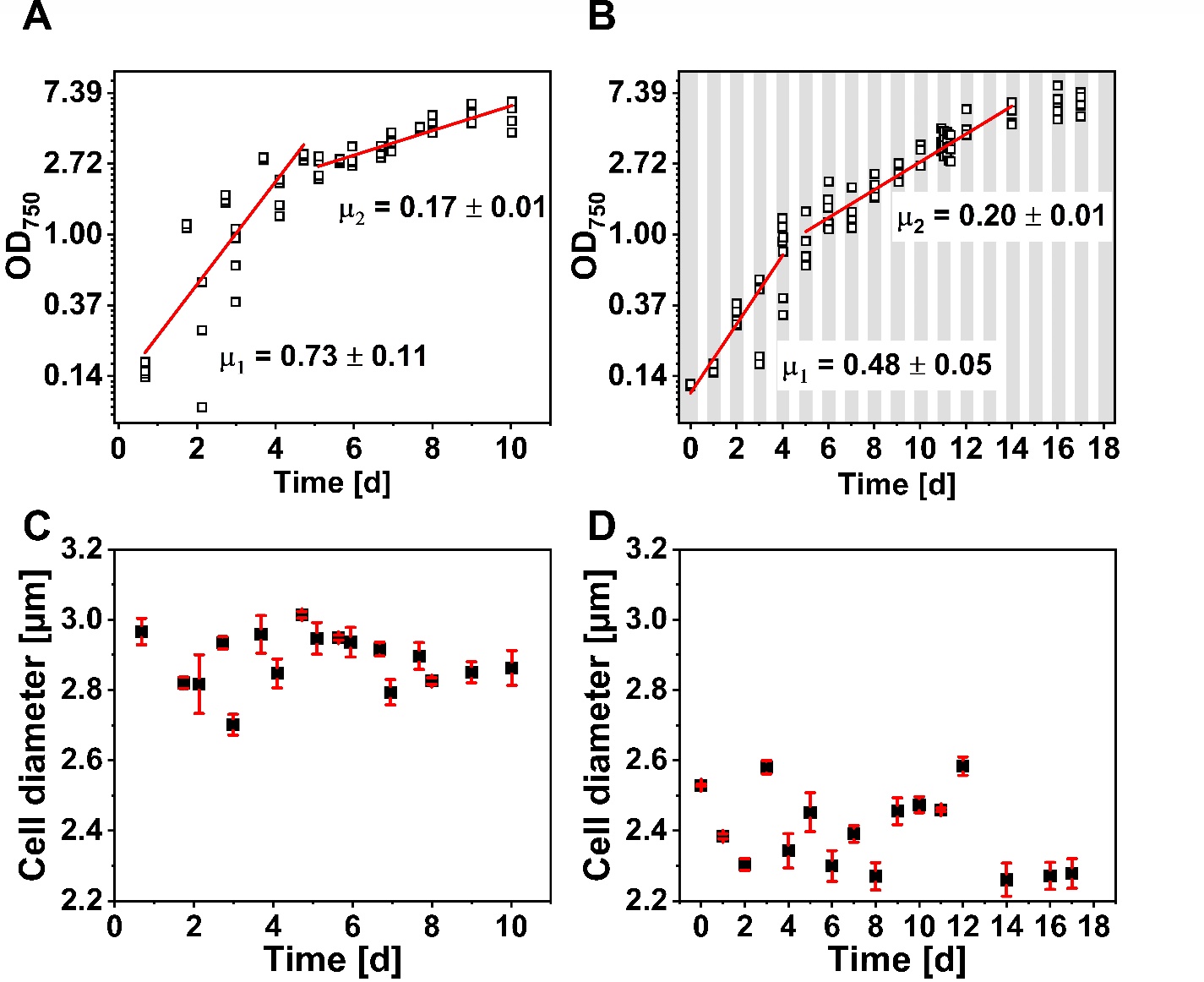


Fig. S1: Growth and cell size of *Synechocystis* cultivated under different illumination cycles. Cells were (A & C) illuminated continuously to obtain *lush* cells or (B & D) illuminated in a 10 h light – 14 h dark cycle to obtain *lean* cells. Upper panels show the semi-logarithmic plot of OD_750_ and lower panels depict the average cell diameter over the cultivation period. Liquid batch cultures were grown in 50 ml nBG11 at 150 rpm, 30°C, 75% relative humidity, 50 µmol_photons_ m^-2^ s^-1^ and ambient CO_2_ levels. Growth rates (µ) were determined by the slope of a linear fit for the semi-logarithmic plots of day 1-4 and day 5-10 (A, n = 8) or 5-13 (B, n = 10). Error bars indicate the standard deviation between biological replicates. White and gray backgrounds indicate illuminated and dark phases, respectively.


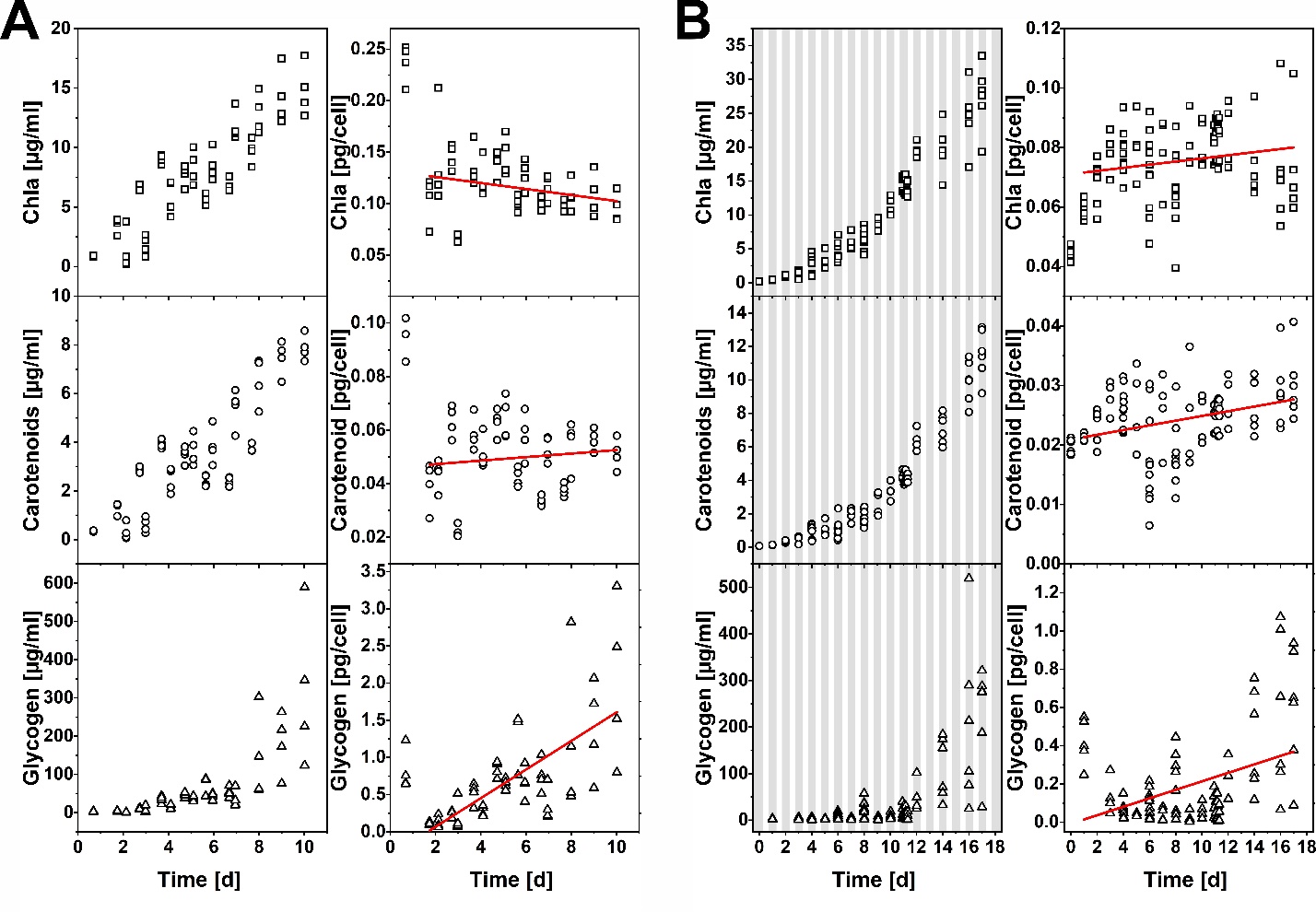


Fig. S2: Chlorophyll, carotenoid and glycogen content of Synechocystis liquid cultures. Cells were (A) illuminated continuously to obtain *lush* cells or (B) illuminated in a 10 h light – 14 h dark cycle to obtain *lean* cells. Liquid cultures were grown in 50 ml nBG11 batch cultures at 150 rpm, 30°C, 75% relative humidity, 50 µmol_photons_ m^-2^ s^-1^ and ambient CO_2_ levels. Concentrations for total culture volume (left panel) and normalized amounts per cell number (right panel) are plotted as a function of time. Red lines show linear fits of the measured data (with A: n=8 and B: n=10).


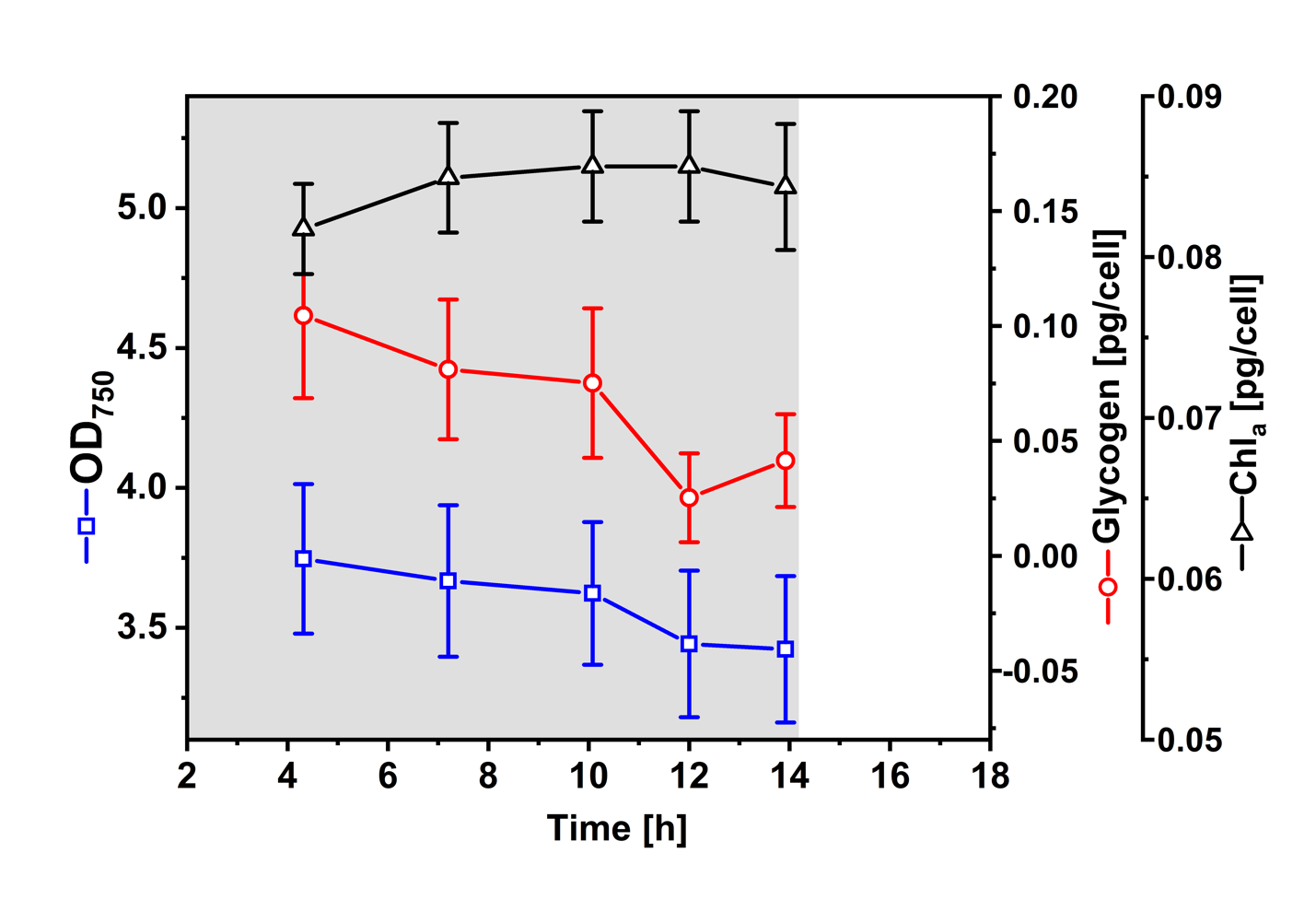
Fig. S3: Growth, Chl_A_ and glycogen content of *Synechocystis* *lean* cells during the dark phase. Progression of OD_750_ (blue boxes), cellular Chl_A_ (black triangles) and glycogen (red circles) content over the period of one dark phase after 11 days of growth. White and grey background indicates the illuminated and the dark phase respectively. Error bars indicate standard deviations (n = 4).


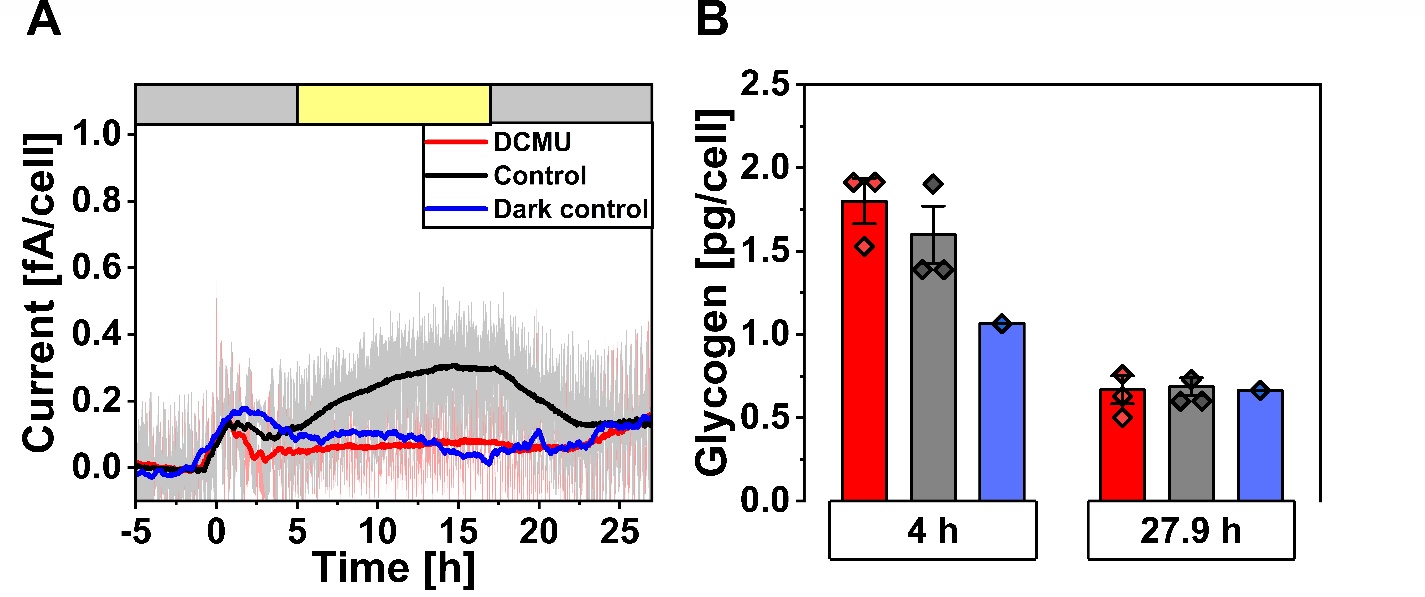


Fig. S4: BPV light current production and in the BPV system without mediator under standard conditions. Chronoamperometry measurements (A) with *lush* *Synechocystis* cells in nBG11 media at an applied potential of 0.5 V against the reference electrode. The headspace of the reactors was flushed with air (30 ml min^−1^). Cells were inoculated at 0 h. Red, black, and blue lines represent the smoothed average of independent measurements (n = 3,3,1 respectively), with the standard error in the respective color. The yellow and gray bars at the top represent illuminated (50 µmol_photons_ m^-2^ s^-1^, cold white LEDs) and dark phases, respectively. Dark controls were not illuminated. Glycogen samples (B) for *lush* cultures were taken at the beginning of the light phase and 24 h later, error bars show the standard deviation between replicates with datapoints plotted on top of bars.


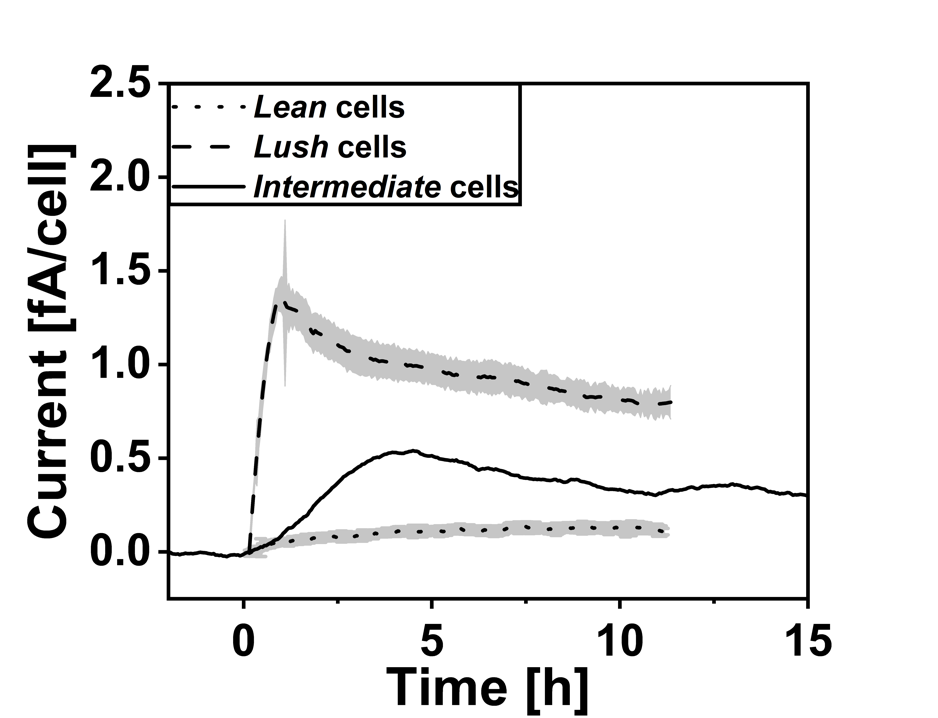


Fig. S5: BPV dark current production under carbon and O_2_ limited conditions. Chronoamperometry measurements with *lean*, *intermediate,* and *lush* *Synechocystis* cells in carbon-reduced nBG11 media with 1 mM ferricyanide as mediator at an applied potential of 0.5 V against the reference electrode. The headspace of reactors was flushed with N_2_ (30 ml min^−1^). Cells were inoculated at 0 h. The depicted data shows only unilluminated conditions in the BPV system. Data for *lean* and *lush* cells show the average of 6 independent measurements each with the standard error as shaded area; data for *intermediate* cells shows one replicate.


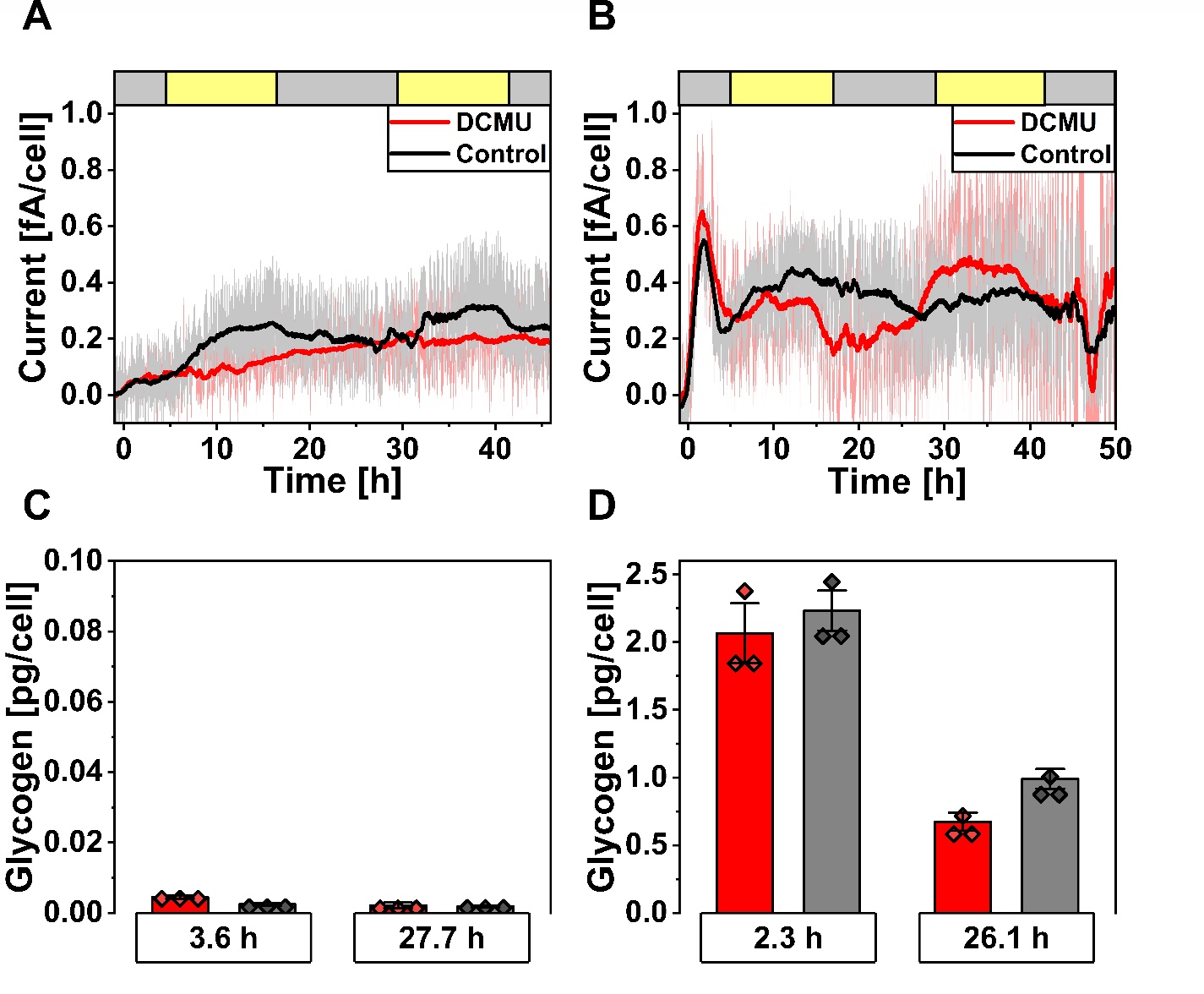
Fig. S6: BPV light current production under carbon and O_2_ limited conditions. Chronoamperometry measurements with *lean* (A) and *lush* (B) *Synechocystis* cells in carbon-reduced nBG11 media with 1 mM ferricyanide as mediator at an applied potential of 0.5 V against the reference electrode. The headspace of reactors was flushed with N_2_ (30 ml min^−1^). Cells were inoculated at 0 h. Red and black lines represent the smoothed average of 3 independent measurements each with the standard error in the respective color. The yellow and gray bars at the top represent illuminated (50 µmol_photons_ m^-2^ s^-1^, cold white LEDs) and dark phases, respectively. Glycogen samples for *lean* (C) and *lush* (D) cultures were taken at the inoculation and at the beginning of each light phase, error bars show the standard deviation between replicates with datapoints plotted on top of bars.


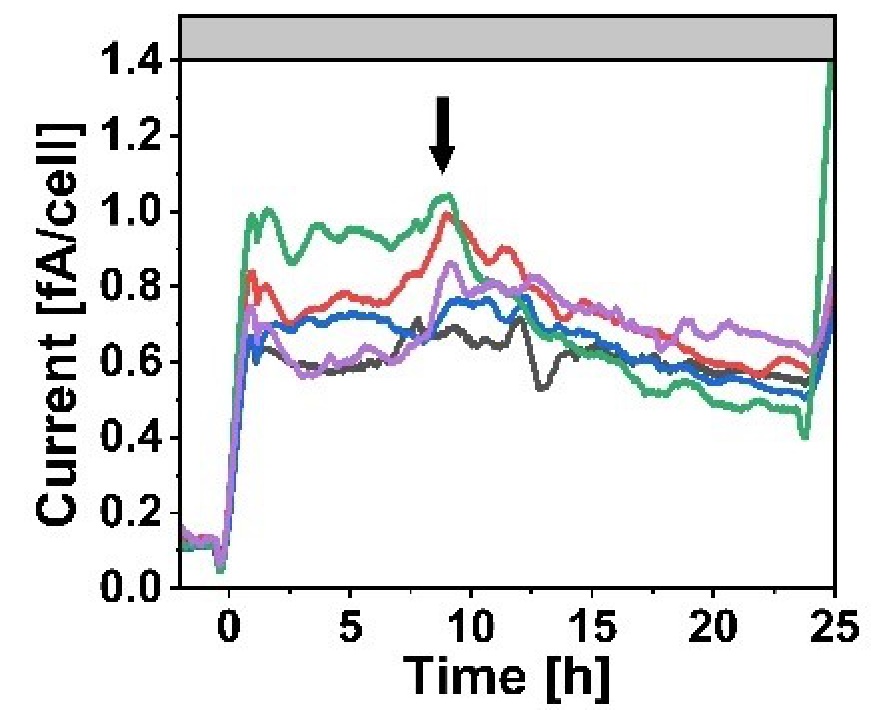


Fig. S7: Chronoamperometry measurements with *lush* *Synechocystis* cells with the addition of the RETC inhibitor rotenone. BPV reactors were kept in dark phase and contain nBG11 media with 1 mM ferricyanide as mediator at an applied potential of 0.5 V against the reference electrode. The headspace of reactors was flushed with air (30 ml min^−1^). Cells were inoculated at 0 h, Rotenone was added 8.5 h later (indicated by black arrow). Each line represents one independent biological replicate.

Table S1: Constituents and concentrations in BG11, nBG11, and carbon-reduced nBG11 media.

| Compounds | Concentration [mg/L] | | |
| --- | --- | --- | --- |
|  | BG11 | nBG11 | carbon-reduced nBG11 |
| Na_2_EDTA | 1.04 | 11.04 | 11.04 |
| Ferric ammonium citrate | 6.00 | 6.00 |  |
| FeCl_3_ · 6H_2_O |  |  | 6.20 |
| Citric acid | 6.00 | 6.00 |  |
| K_2_HPO_4_ | 40 | 40 | 40 |
| NaNO_3_ | 1500 | 1500 | 1500 |
| CaCl_2_ · 2H_2_O | 36 | 36 | 36 |
| MgSO_4_ · 7H_2_O | 75 | 75 | 75 |
| Na_2_CO_3_ | 20 | 20 |  |
| ZnSO_4_ · 7H_2_O | 0.22 | 0.22 | 0.22 |
| H_3_BO_3_ | 2.86 | 2.86 | 2.86 |
| MnCl_2_ · 4H_2_O | 1.81 | 0.54 | 0.54 |
| CoCl_2_ · 6H_2_O | 0.05 | 0.05 | 0.05 |
| CuSO_4_ · 5H_2_O | 0.08 | 0.08 | 0.08 |
| NaMoO_4_ · 2H_2_O | 0.39 | 0.39 | 0.39 |
| HEPES buffer (pH= 8) | 2.38 |  |  |
| Agar | 7500 |  |  |
| Na_2_S_2_O_3_ | 0.3 % (w/v) |  |  |
